# Light-Dependent Expression and Promoter Methylation of the Genes Encoding Succinate Dehydrogenase, Fumarase, and NAD-Malate Dehydrogenase in Maize (*Zea mays* L.) Leaves

**DOI:** 10.3390/ijms241210211

**Published:** 2023-06-16

**Authors:** Alexander T. Eprintsev, Dmitry N. Fedorin, Abir U. Igamberdiev

**Affiliations:** 1 Department of Biochemistry and Cell Physiology, Voronezh State University, 394018 Voronezh, Russia; bc366@bio.vsu.ru (A.T.E.); rybolov@mail.ru (D.N.F.); 2 Department of Biology, Memorial University of Newfoundland, St. John’s, NL A1C 5S7, Canada

**Keywords:** DNA methylation, mitochondria, phytochrome, fumarase, NAD-malate dehydrogenase, succinate dehydrogenase, tricarboxylic acid cycle, *Zea mays*

## Abstract

The expression and methylation of promoters of the genes encoding succinate dehydrogenase, fumarase, and NAD-malate dehydrogenase in maize (*Zea mays* L.) leaves depending on the light regime were studied. The genes encoding the catalytic subunits of succinate dehydrogenase showed suppression of expression upon irradiation by red light, which was abolished by far-red light. This was accompanied by an increase in promoter methylation of the gene *Sdh1-2* encoding the flavoprotein subunit A, while methylation was low for *Sdh2-3* encoding the iron-sulfur subunit B under all conditions. The expression of *Sdh3-1* and *Sdh4* encoding the anchoring subunits C and D was not affected by red light. The expression of *Fum1* encoding the mitochondrial form of fumarase was regulated by red and far-red light via methylation of its promoter. Only one gene encoding the mitochondrial NAD-malate dehydrogenase gene (*mMdh1*) was regulated by red and far-red light, while the second gene (*mMdh2*) did not respond to irradiation, and neither gene was controlled by promoter methylation. It is concluded that the dicarboxylic branch of the tricarboxylic acid cycle is regulated by light via the phytochrome mechanism, and promoter methylation is involved with the flavoprotein subunit of succinate dehydrogenase and the mitochondrial fumarase.

## 1. Introduction

The interaction between photosynthesis and mitochondrial respiration represents the central crossing point in the network of metabolic regulation in photosynthetic tissues. Many studies starting from the middle of the 20th century [1] clearly demonstrated that the intensity of mitochondrial respiration is decreased in light [2,3]. While many respiratory enzymes are regulated directly via changes in redox balance and concentrations of photosynthetic and respiratory intermediates [4,5,6], the long-term effects of the light regime occur at the transcriptional level through the regulation of expression of the genes encoding respiratory enzymes.

Light-dependent control of the metabolism of di- and tricarboxylic acids in plant leaves is commonly associated with active forms of phytochrome [7,8,9] and cryptochrome [10,11]. This regulatory mechanism is associated with the rearrangement of metabolic flows of di- and tricarboxylic acids when plants are exposed to light of different intensities and spectral compositions [12]. In light, the tricarboxylic acid (TCA) cycle is transformed into the non-cyclic mode, resulting in citrate export to the cytosol [13,14,15]. The dicarboxylic branch of the TCA cycle, which includes the reactions catalyzed by succinate dehydrogenase, fumarase, and malate dehydrogenase, is important for the exchange of redox equivalents between the mitochondria and extramitochondrial compartments, as well as for the regulation of redox levels inside the mitochondria [6,16].

Light signaling occurs at different levels of metabolic organization and includes fine mechanisms of epigenetic control of gene expression [17], which is realized via changes in the degree of DNA methylation and interaction with transcription factors [18,19]. The presence of specific *cis*-regulatory regions in promoters of the genes encoding photosynthetic and respiratory enzymes indicates an important role of transcription factors in phytochrome-dependent gene regulation via DNA methylation-demethylation [20]. In previous studies, we described phytochrome-dependent epigenetic regulation of citrate synthase [21] and aconitase [22] operating in the tricarboxylic branch of the TCA cycle. Light-dependent regulation of expression of the enzymes representing the dicarboxylic branch of the TCA cycle has also been demonstrated [8,11,23], and it is important to establish the role of epigenetic mechanisms in this process.

Succinate dehydrogenase (SDH; EC 1.3.99.1) is the only enzyme of the TCA cycle that is incorporated into the inner mitochondrial membrane and functions as part of the mitochondrial electron transport chain (ETC). SDH is an oligomeric protein consisting of four main subunits, A (flavoprotein), B (iron-sulfur protein), C and D (anchoring proteins), and accessory plant-specific subunits, the functions of which are not well defined [24]. The subunits of SDH are genetically determined by several genes, including *Sdh1-2* (LOC100279930) encoding subunit A, *Sdh2-3* (LOC109944584) encoding subunit B, *Sdh3-1* (LOC100283351) encoding subunit C, and the only gene *Sdh4* (LOC100280324) encoding subunit D [24]. The *Sdh1-2* and *Sdh2-3* genes encoding the subunits of the SDH catalytic dimer have the highest expression in green leaves of maize seedlings relative to other SDH catalytic dimer genes [24]. Fumarase (EC 4.2.1.2) catalyzes the reversible hydration of fumarate, formed by SDH, to malate in the TCA cycle. It is represented by the mitochondrial and cytosolic forms encoded by different genes: *Fum1* (LOC103652742) and *Fum2* (LOC103633973) [11,25]. NAD-dependent malate dehydrogenase (MDH; EC 1.1.1.37) is represented by several molecular forms in plant cells, which are associated with different compartments [26]. High MDH activity establishes the equilibrium of NAD^+^ and NADH [27]. The mitochondrial forms of MDH encoded by the genes *mMdh1* (LOC100274264 in maize) and *mMdh2* (LOC100273428) mediate several processes by catalyzing the reaction of interconversion of malate and oxaloacetate in the TCA cycle [28,29].

In this study, we irradiated maize (*Zea mays* L.) plants with light at wavelengths that are specifically captured by phytochromes, and we monitored the activity, expression, and promoter methylation of the genes encoding the four subunits of SDH and the mitochondrial isoforms of fumarase and MDH in the leaves of plants kept in darkness and upon irradiation. We conclude that the dicarboxylic branch of the TCA cycle is regulated by light via phytochrome signaling, and the epigenetic mechanism that involves promoter methylation is responsible for the regulation of expression of the flavoprotein subunit of succinate dehydrogenase and the mitochondrial fumarase.

## 2. Results

### 2.1. Activities of Succinate Dehydrogenase, Fumarase, and NAD-Malate Dehydrogenase

**Figure 1:**
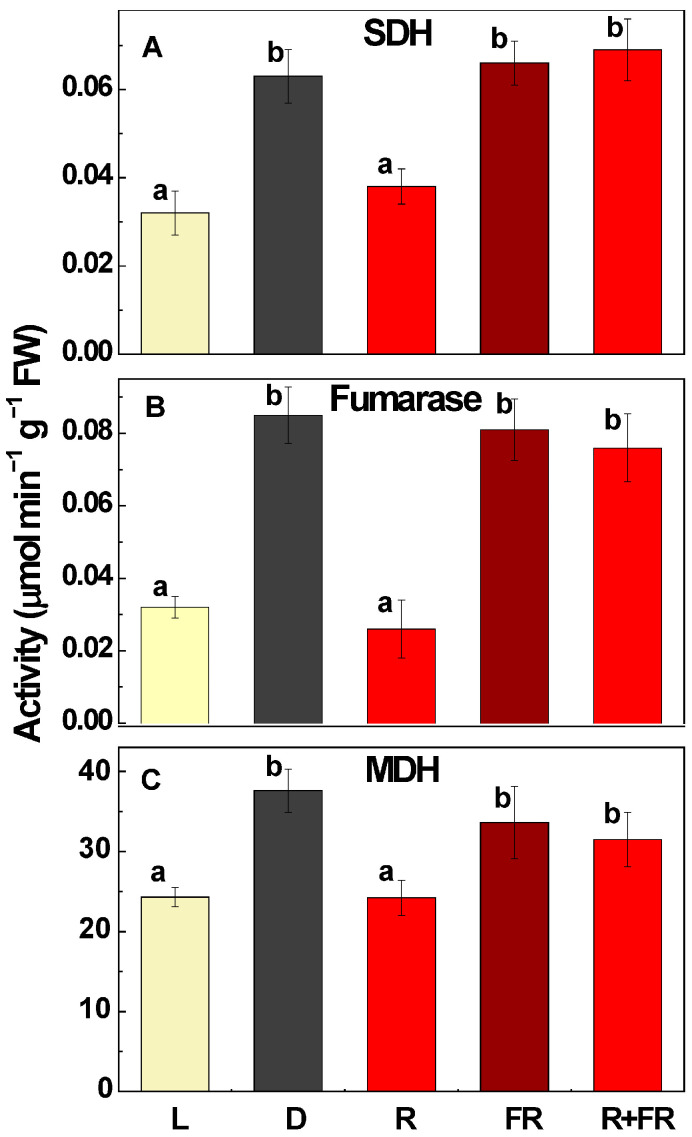
Changes in the activities of succinate dehydrogenase (SDH) (**A**), mitochondrial fumarase (**B**), and mitochondrial NAD-malate dehydrogenase (MDH) (**C**) in maize leaves under different lighting conditions. L, plants illuminated with white light; D, plants kept in darkness; R, plants illuminated with red (660 nm) light; FR, plants illuminated with far-red (730 nm) light; R + FR, plants illuminated with red (660 nm) light followed by far-red (730 nm) light. Data represent the means of three biological repeats ± SD. Significant differences at *p* < 0.05 are indicated by different letters.

The activities of SDH and the mitochondrial forms of fumarase and MDH were dependent on the light regime (Figure 1). SDH activity was twice as high in plants kept in darkness as in plants under light. It was suppressed by red light and exhibited the same level as in plants kept in darkness if the plants were irradiated by far-red light (applied to plants kept in darkness or after red light irradiation). The mitochondrial fumarase activity exhibited a similar profile as SDH, with an even higher (3-fold) difference between plants kept in darkness and plants illuminated by white or red light. For the mitochondrial MDH, the profile of activity was also similar, although the difference was lower (~1.5-fold). It is important to note that the activity of MDH was three orders of magnitude higher than the activities of SDH and fumarase (Figure 1).

### 2.2. CpG Dinucleotides in the Genes Encoding Succinate Dehydrogenase Subunits and the Mitochondrial Isoforms of Fumarase and NAD-Malate Dehydrogenase

**Figure 2:**
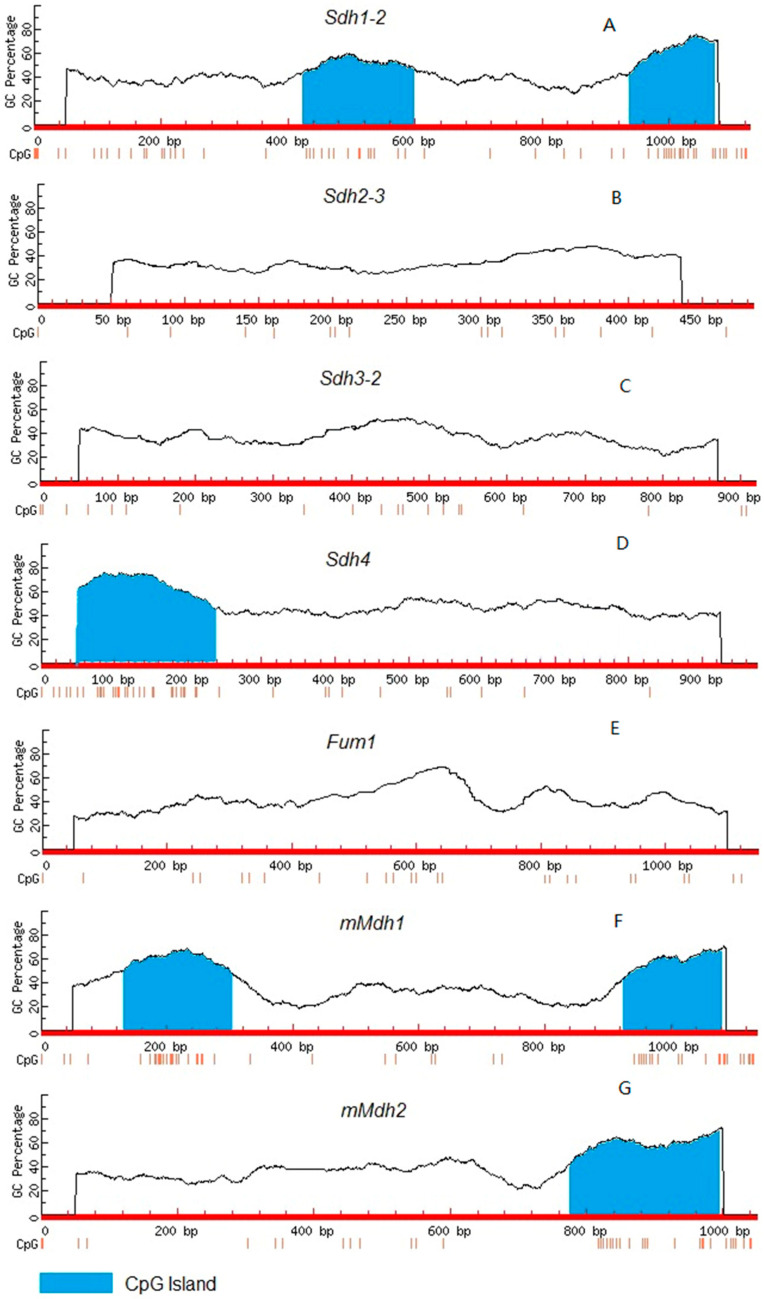
Analysis of CG dinucleotides in the promoters of the genes encoding succinate dehydrogenase subunits (**A**) (*Sdh1-2*), (**B**) (*Sdh2-3*), (**C**) (*Sdh3-1*), (**D**) (*Sdh4*), mitochondrial fumarase (**E**) (*Fum1*), and two isoforms of mitochondrial NAD-malate dehydrogenase ((**F**) *mMdh1* and (**G**) *mMdh2*) in maize. The positions of CG nucleotides are indicated by vertical lines. The blue color denotes promoter regions that are CpG islands.

The study of the promoter structure of the maize SDH flavoprotein gene *Sdh1-2* revealed the uneven distribution of CG dinucleotides in its nucleotide sequence. Two CpG islands were found in the *Sdh1-2* gene promoter, indicating the possibility of its regulation upon changes in the methylation status of CG dinucleotides forming the CpG islands. The first island’s position was located from −354 to −527, and the second island’s position was located from −867 to −1000 (Figure 2A). The study of the nucleotide sequence of the promoter of the gene *Sdh2-3* showed the absence of CpG islands in its composition (Figure 2B). The promoter of the *Sdh3-1* gene had a low content of CG dinucleotides in its composition (Figure 2C), while the promoter of the *Sdh4* gene contained one CpG island (Figure 2D).

The promoter of the gene *Fum1* encoding the mitochondrial fumarase did not contain CpG islands, and the individual CG dinucleotides were distributed relatively evenly over the whole nucleotide sequence of the promoter (Figure 2E). The promoter of the gene *mMdh1* contained two CpG islands located at its ends, while the promoter of the gene *mMdh2* contained one CpG island (Figure 2F,G).

### 2.3. Expression of Succinate Dehydrogenase Genes

**Figure 3:**
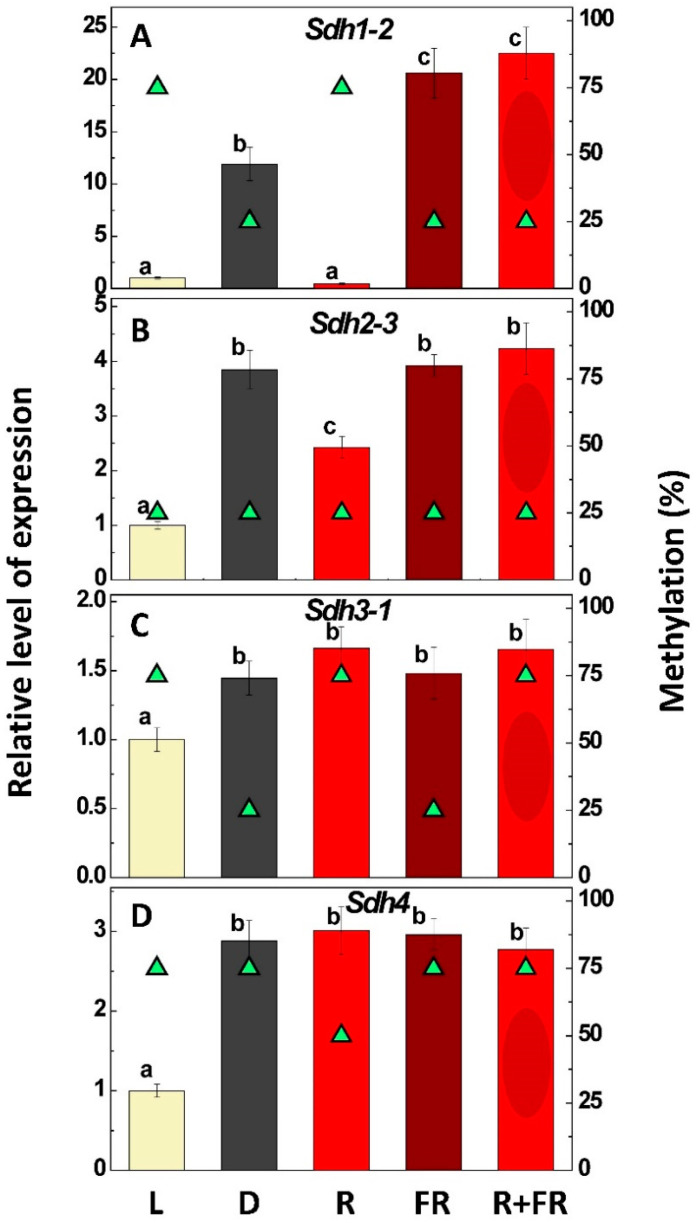
Relative levels of expression of succinate dehydrogenase genes *Sdh1-1* (**A**), *Sdh2-3* (**B**), *Sdh3-1* (**C**), and *Sdh4* (**D**) (columns) and the degree of methylation of their promoters (green triangles) in maize leaves under different lighting conditions. L, plants illuminated with white light; D, plants kept in darkness; R, plants illuminated with red (660 nm) light; FR, plants illuminated with far-red (730 nm) light; R + FR, plants illuminated with red (660 nm) light followed by far-red (730 nm) light. Data represent the means of three biological repeats ± SD. Significant differences at *p* < 0.05 are indicated by different letters.

All investigated genes (*Sdh1-2, Sdh2-3, Sdh3-1, Sdh4*) encoding the four SDH subunits A, B, C, and D exhibited lower expression in plants under light than in those kept in darkness (Figure 3). However, only the genes encoding the catalytic subunits were regulated by red and far-red light. Red and far-red light were ineffective in the regulation of the anchoring subunits C and D. The strongest effect of light (white and red) was observed for the gene *Sdh1-2* encoding the flavoprotein subunit A, for which expression was decreased more than ten times, and this was accompanied by a change in the methylation status of its promoter from 25% to 75% (Figure 3A). For the gene *Sdh2-3* encoding the iron-sulfur subunit B, the decreases were almost 4-fold in plants under white light and 2-fold in plants upon red light irradiation, and these changes were not accompanied by changes in promoter methylation (Figure 3B).

Although variations in promoter methylation were observed for the gene *Sdh3-1* encoding the anchoring subunit C of SDH, they could have been related to the effect of white light but not to the flash of red light (Figure 3C). While methylation decreased upon irradiation by far-red light, this was not accompanied by changes in expression of the gene *Sdh3-1*. For the anchoring subunit D encoded by *Sdh4*, some decrease in promoter methylation was observed only upon irradiation by red light, without changes in expression (Figure 3D).

### 2.4. Expression of the Mitochondrial Fumarase Gene

**Figure 4:**
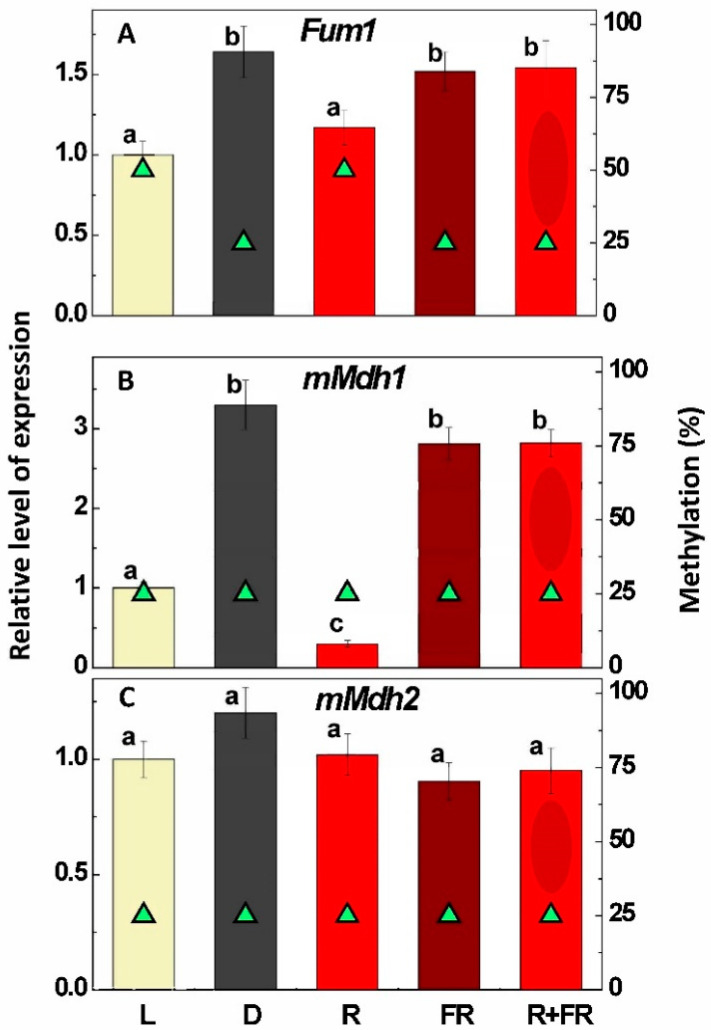
Relative levels of expression of the mitochondrial fumarase gene *Fum1* (**A**) and two NAD-malate dehydrogenase genes *mMdh1* (**B**) and *mMdh2* (**C**) (columns) and the degrees of methylation of their promoters (green triangles) in maize leaves under different lighting conditions. L, plants illuminated with white light; D, plants kept in darkness; R, plants illuminated with red (660 nm) light; FR, plants illuminated with far-red (730 nm) light; R + FR, plants illuminated with red (660 nm) light followed by far-red (730 nm) light. Data represent the means of three biological repeats ± SD. Significant differences at *p* < 0.05 are indicated by different letters.

Although expression of the gene *Fum1* encoding the mitochondrial form of fumarase was decreased in plants under light and upon irradiation by red light by only 30–40%, this change was clearly under control of promoter methylation, which changed from 25% to 50% following incubation of the plants in light or upon their irradiation by red light (Figure 4A). Even a moderate change in expression of the gene *Fum1* resulted in a major (3-4-fold) decrease in fumarase activity.

### 2.5. Expression of the Genes Encoding Mitochondrial NAD-Malate Dehydrogenases

The study of the two genes *mMdh1* and *mMdh2* encoding the mitochondrial isoenzymes of MDH revealed that only *mMdh1* was regulated by light and responded to red and far-red irradiation (Figure 4B,C). The response of *mMdh1* was quite significant (three times lower expression under white light and almost ten times lower expression upon far-red light irradiation); however, no promoter methylation was involved in this regulation. The level of promoter methylation of the gene *mMdh1* remained low (25%) under all conditions, and the same was observed for the gene *mMdh2,* which, contrary to *mMdh1*, did not exhibit a response to light or irradiation by red/far-red light (Figure 4C).

## 3. Discussion

The regulation of plant metabolism by light has been studied for many years [1]; however, it has only been assessed recently in terms of distinguishing the mechanisms involved, including the rearrangement of transcriptional networks that mediate photoreceptor signals [30,31,32]. Epigenetic mechanisms are directly involved in the control of light-responsive genes via modulation of their binding to transcription factors [33,34]. Analysis of the nucleotide sequences of promoters of the genes encoding respiratory enzymes indicated the presence of specific binding sites in their structures for transcription factors of the basic helix-loop-helix (bHLH) family, among them the phytochrome-interacting factors (PIFs) that mediate the action of phytochrome. DNA methylation, which includes large-scale cytidylate methylation and less abundant adenylate methylation [21], plays an important role in the regulation of a wide range of processes in plants [35], e.g., it participates in the response to various stress conditions [36,37,38]. However, only a few studies elucidating the changes in DNA methylation in plants in response to light have been performed to date. Maize plants contain phytochromes A, B, and C, which are encoded by pairs of genes (homeologs), and they do not have phytochromes D and E; however, the expression of *PhyA1*, *PhyB1*, and *PhyC1* prevails in all tissues of maize seedlings [39,40]. In the current work, we used wavelengths of red and far-red light that regulate all types of phytochromes and did not specify which phytochromes were involved in the regulation of the investigated enzymes.

The enzymes investigated in this study belong to the dicarboxylic branch of the TCA cycle [6]. Their regulation is important for the adaptation of plant metabolism to light and switching the TCA cycle from the complete to open mode in which malate is actively exchanged between the mitochondria and cytosol and citrate is exported for biosynthetic purposes [14,15]. While the previous studies showed that SDH and fumarase are regulated by light via thioredoxin [41], it was also demonstrated that SDH, fumarase, and MDH are regulated by light at the transcriptional level (reviewed in [9]). This regulation results in the inhibition of SDH [8] and the mitochondrial form of fumarase, while the cytosolic form of fumarase is unaffected [42]. It was shown earlier that the phytochrome system differentially regulates the isoforms of enzymes participating in the corresponding processes related to photosynthesis and heterotrophic respiration, such as the cytosolic and chloroplast forms of glyceraldehyde phosphate dehydrogenase [43]. A similar pattern was observed for the mitochondrial and cytosolic forms of TCA cycle enzymes [9,42,44].

The current study demonstrates that light regulation of SDH and the mitochondrial forms of fumarase and MDH is under control of the phytochrome mechanism. The activities of all three enzymes, being inhibited in plants under light as compared to those kept in darkness, were also suppressed by the flash of red light, which was reversed by far-red light (Figure 1). The levels of light inhibition were different for SDH (about 50% suppression in maize leaves), fumarase (3-4-fold suppression), and MDH (~1.5-fold suppression). Further research conducted in this study showed that mechanisms based on promoter methylation may be involved, although they cannot explain all of the transcriptional changes observed for the genes encoding respiratory enzymes.

SDH contains two catalytic subunits, two anchoring subunits common to plant and animal mitochondria, and several plant-specific subunits [23]. In this study, we investigated expression of the gene *Sdh1-2* encoding the flavoprotein catalytic subunit, the gene *Sdh2-3* encoding the iron-sulfur subunit, and the genes *Sdh3-1* and *Sdh4* encoding the anchoring subunits. The choice of the genes *Sdh1-2, Sdh2-3*, and *Sdh3-1* was based on the study of their higher expression in maize leaves as compared to other genes encoding corresponding subunits [45]. The genes *Sdh1-1*, *Sdh2-1*, and *Sdh2-2* are actively expressed mostly in the early stages of development (early germination), e.g., in the scutella of germinating maize seeds [45]. This allowed us to concentrate on the expression of the studied genes.

As seen from Figure 3A–D, phytochrome-mediated regulation was evident only for the catalytic subunits of SDH and was stronger for the flavoprotein subunit A, for which the gene *Sdh1-2* was suppressed one order of magnitude less upon red light treatment while far-red light activated its expression above the level observed in plants kept in darkness. This indicated that *Sdh1-2* expression is under strong control of the phytochrome system and promoter methylation represents the mechanism responsible for its regulation. This becomes possible, in particular, because the gene *Sdh1-2* contains two expanded CpG islands (Figure 2A), which appear to be the targets of cytosine methylation. The increased level of promoter methylation of the gene *Sdh1-2* led to a marked decrease in the content of its transcripts in plants under light and upon red light irradiation. Expression of the gene *Sdh2-3* encoding the iron-sulfur subunit B is also under control of the phytochrome system, but this regulation is more moderate. Red light decreased the expression of *Sdh2-3* less than 2-fold as compared to plants kept in darkness, and this suppression was not as strong as the decrease in expression observed in plants grown in light. The obtained data clearly show that the regulation of expression of subunit B is not related to changes in the methylation of promoters of the gene *Sdh2-3*, which remained constantly low independent of the light regime (Figure 3B). This may be explained by the absence of CpG islands in the promoter of the gene *Sdh2-3* (Figure 2B).

Although expression of the genes encoding the two anchoring subunits (C and D) was partially suppressed in maize plants grown in light, this process is not regulated via phytochrome. In plants kept in darkness and upon far-red light irradiation, the promoter of *Sdh3-1* was less methylated, but this did not affect the expression as compared to that in plants upon red light irradiation (Figure 3C). It was shown earlier that expression of subunit C during germination is regulated by the level of methylation [46]. In the case of regulation by light, the promoter of *Sdh3-1* in plants grown in darkness was less methylated, but the phytochrome mechanism was not involved. It is possible that different mechanisms of transmission of light signals use other photoreceptors in this case. The promoter of *Sdh3-1* did not contain a CpG island (Figure 2C), but there were few CpG dinucleotides that could undergo methylation. The presence of a CpG island in the promoter of *Sdh4* gene favored its possible regulation via methylation, but there was no evidence of this in the current study. Previously it was shown that the pigment cryptochrome, which perceives blue wavelengths, is involved in the regulation of SDH and fumarase [11]. Its possible involvement in the regulation of expression of the genes encoding the anchoring subunits will be a matter of future studies of SDH regulation.

The obtained data demonstrate that the mitochondrial form of fumarase is also regulated via methylation of the promoter of its gene *Fum1*. Smaller changes (less than 2-fold) in expression as compared to *Sdh1-2* caused significant changes in activity (3- to 4-fold). Probably only moderate changes in expression were associated with the low abundance of CpG dinucleotides in the *Fum1* gene and the absence of CpG islands (Figure 2E). Nevertheless, even these moderate alterations in expression corresponded to significant changes in fumarase activity, which was strongly inhibited in light. The phytochrome mechanism is involved in this process, since red light led to higher promoter methylation and lower expression of *Fum1*, and this process was reversed by far-red light (Figure 4A). Earlier, a clear relationship between the level of fumarase transcripts and the methylation status of individual CG dinucleotides was demonstrated in other physiological conditions (e.g., during germination [44] and in response to salt stress [47]).

Previously we showed that while the mitochondrial form of fumarase is inhibited in light, the cytosolic form is not affected [42], and both processes can be mediated by phytochrome and cryptochrome [11]. Cytosolic processes partially substitute mitochondrial pathways upon irradiation, and this may be part of the general mechanism of regulation of plant respiration in light [6,15,16]. SDH and fumarase represent an essential branch of the TCA cycle that plays a role in switching between the closed (complete) and open (partial) modes of the TCA cycle [6,13,14,16].

It is important to note that light-dependent regulation of SDH and fumarase occurs at different levels, from the transcriptional level that we report in this study to the level of posttranslational modifications [8], including regulation via thioredoxin [41]. The transduction of signals from phytochrome can be involved at different levels. The multilevel regulation of mitochondrial enzymes results in fine-tuned modulation of respiration in light, which includes short- and long-term effects and provides flexibility for the plant metabolic system [48].

A multigene family encodes MDH in plant cells, and different genes encode MDH forms having different subcellular localizations [26]. Two genes encode the mitochondrial forms of MDH in Arabidopsis [28] and three genes have been identified in maize [49], although later investigations suggested that the third gene encodes a mitochondrial-like form that may not be transported to the mitochondria [29]. The results obtained in this study clearly demonstrate that only *mMdh1* is regulated via phytochrome and this regulation is quite strong, while *mMdh2* is expressed in a phytochrome- and light-independent manner. Moderate light-dependent changes in MDH activity in the mitochondrial fraction may be due to the lack of expression changes in *mMdh2*. No methylation is involved in the expression of either gene.

MDH possesses very high activity, which in our study was three orders of magnitude higher than the activities of SDH and fumarase (Figure 1). MDH not only interconverts malate and OAA but also establishes their ratio (which shifts depending on pH) and the ratio of reduced and oxidized NAD [6]. Thus, MDH represents a thermodynamic buffering enzyme [50,51,52]. It is possible that only one MDH isoform has a preferential role in the operation of the TCA cycle, while the other is more tuned to thermodynamic buffering. The latter is important particularly during photorespiratory oxidation of glycine where it keeps the NADH/NAD^+^ ratio low to prevent the inhibition of glycine decarboxylase and provide a high unrestricted photorespiratory flux [53,54]. Our study clearly shows that the genes *mMdh1* and *mMdh2* are regulated in different ways. While *mMdh1* is suppressed by light via phytochrome, *mMdh2* is not responsive to light (Figure 4B,C). The promoter of *mMdh1* contains two CpG islands and the promoter of *mMdh2* contains one CpG island (Figure 2F,G); nevertheless, we could not find any changes in the methylation status of these promoters depending on the light conditions. This means that the phytochrome-dependent regulation of *mMdh1* does not involve promoter methylation, while the absence of light-dependent regulation of *mMdh2* corresponds with the absence of changes in the methylation status of its promoter. Together with changes in NAD-malic enzyme expression and activity depending on light/dark conditions [55,56], the epigenetic regulation of MDH provides an adjustment of malate metabolism in photosynthetic plant cells.

**Figure 5:**
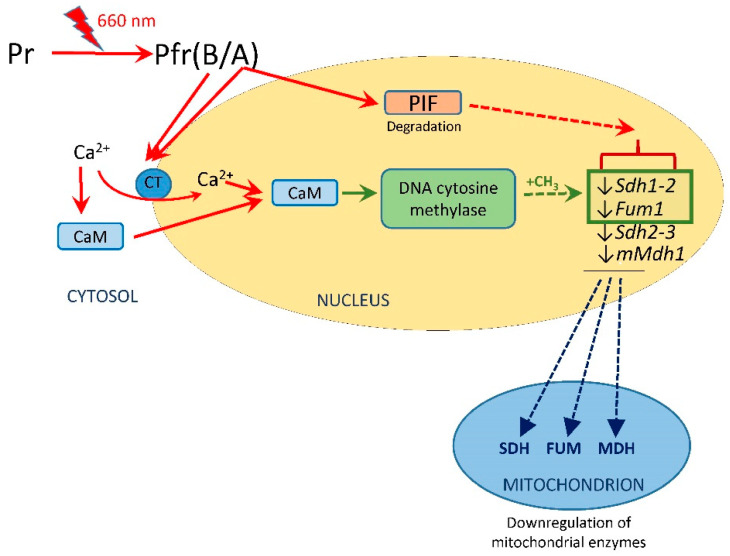
Mechanism of phytochrome-dependent regulation of transcriptional activity of the genes encoding the enzymes of the dicarboxylic acid metabolism. Abbreviations: Pr, inactive form of phytochrome, Pfr, active form of phytochrome A or B; CaM, calmodulin; CT, calcium transporter; FUM, fumarase; MDH, NAD-malate dehydrogenase; SDH, succinate dehydrogenase; PIF, phytochrome-interacting factor. Solid lines—activation or transport, dotted lines—inhibition, ↓—decrease in expression.

The obtained results show that the inhibition of plant respiration can occur at the level of SDH, fumarase, and MDH. which represent the dicarboxylic branch of the TCA cycle. In addition to regulation of the pyruvate dehydrogenase complex and the enzymes of the tricarboxylic branch (reviewed in [6]), this regulation provides a redistribution of the metabolic fluxes of organic acids in photosynthetic plant cells. Regulation of expression of the genes encoding these enzymes is schematically presented in Figure 5. It is shown that the red light signal can be transduced both via PIF and through Ca^2+^ transport to the nucleus in the case of phytochrome A, and only though Ca^2+^ transport to the nucleus in the case of phytochrome B. PIF under the action of phytochrome is rapidly phosphorylated [57,58], resulting in its ubiquitination and proteasome-mediated degradation [59]. Ca^2+^ can penetrate the nuclear membrane either as an ion or after binding with calmodulin (CaM). Binding with CaM can also take place in the nucleus. CaM, in particular CaM7, which is active in the regulation of light-dependent genes [60], acts as a transcriptional regulator that directly interacts with the promoters of several inducible genes by binding to their G- and E-regions [61]. The complex formed by Ca^2+^ with CaM activates DNA cytosine methylase, which results in methylation of the genes *Sdh1-2* and *Fum1*. The genes *Sdh2-3* and *mMdh1* are suppressed by a mechanism that does not involve promoter methylation.

## 4. Material and Methods

### 4.1. Object of Investigation

Leaves of 14-day-old maize plants (*Zea mays* L., cv Voronezhskaya-76 obtained from the Voronezh branch of the All-Russian Research Institute of Maize), grown hydroponically in 12 h daylight at an intensity of 90 µmol quanta m^−2^ s^−1^, were used in this study. White light was emitted by fluorescent lamps (growth setup Flora-1, PhytoSun, Moscow, Russia). Irradiation by red and far-red light was performed using LEDs with an emission regions of 640–680 nm (KIPD40M40-K-P6, Kaskad-Elektro, Moscow, Russia) and 710–750 nm (ZL127A-5, Kaskad-Elektro, Moscow, Russia), respectively. The intensity of red or far-red light during irradiation was 4 µmol quanta m^−2^ s^−1^ and the irradiation lasted 15 min, which was sufficient for the initiation of signal reactions by the phytochrome system but did not lead to intensification of photosynthesis [8].

### 4.2. Determination of Enzymatic Activities

The measurement of enzymatic activities was performed in the organellar (enriched by mitochondria) fractions of maize leaves. All procedures took place at 4 °C. Maize leaves (2 g) were homogenized in 10 mL of 100 mM potassium phosphate buffer, pH 7.6, containing 0.3 M sucrose and 1 mM ethylenediaminetetraacetic acid (EDTA), and filtered through four layers of cheesecloth. After the first centrifugation at 1300× *g* for 5 min, the debris from cell walls was discarded and the supernatant was centrifuged again at 14,000× *g* for 20 min. The pellet containing the mitochondria (contaminated with peroxisomes and other organelles) was ruptured in 5 mL of 50 mM Tris-HCl buffer, pH 7.5, containing 1 mM EDTA, 10 mM KCl, 1 mM MgCl_2_, and 0.01% Tween 80, and centrifuged at 14,000× *g* for 10 min. The supernatant was used for the determination of enzymatic activities, which was performed using an SF-2000 spectrophotometer (OKB Spectr, St. Petersburg, Russia).

The activity of succinate dehydrogenase (EC 1.3.5.1) was measured at 600 nm using the artificial electron acceptor dichlorophenolindophenol (DCPIP) in medium containing 30 mM potassium phosphate buffer, pH 7.8, 1 mM phenazine methosulfate (PMS), 0.08 mM DCPIP, 2 mM sodium azide, and 20 mM sodium succinate [62]. The activity of fumarase (EC 4.2.1.2) was measured by an increase in optical density at 240 nm at 25 °C due to the formation of the double bond in the fumarate molecule. The assay medium contained 50 mM potassium phosphate, pH 7.0, 50 mM malate, and 5 mM MgCl_2_ [63]. The activity of NAD-malate dehydrogenase (EC 1.1.1.37) was detected at 340 nm in 50 mM HEPES, pH 7.4, by the oxidation of 0.2 mM NADH in the presence of 2 mM OAA [64]. The chemicals were obtained from Sigma-Aldrich (St. Louis, MO, USA). All activities were calculated per gram of fresh weight of leaves. The units of enzymatic activity corresponded to the formation of 1 µmol product per minute at 25 °C.

### 4.3. RNA Isolation and RT-PCR

The total RNA was isolated from maize leaves by guanidinum thiocyanate–phenol–chloroform extraction according to Chomczynski and Sacchi [65]. Polymerase chain reaction with gene-specific primers was performed using AmpliSens reagent (Helicon, Moscow, Russia) [24]. The primers for PCR analysis had the following nucleotide sequences: *Sdh1-2* forward—5′-CGAATGGGTCATTGCCAACT-3′, reverse—5′-ACCTTTGAAAGGGTACAAAA-3′; *Sdh2-3* forward—5′-GAGAGGCTACAGGCAATAACTGAG-3′, reverse—5′-GGATTTTGACTTGCATGGGATTG-3′; *Sdh3-1* forward—5′-AAGGAGGCTTCTCCATCTCC-3′, reverse—5′-CAGAGCTGCTACAGGGGAAG-3′; *Sdh4* forward—5′-TTCGGCCATTACGGTCCGGAAG-3′, reverse—5′-AGCGGAATGAAATCTTGAGGA-3′; *Fum1* forward—5′-GATTACTTCGATCATTGAGGT-3′, reverse—5′-ACCAGAACTCGCGGATGTGGC-3′; *mMdh1* forward—5′-TATGCTGGTGCTGTTTTTGC-3′, reverse—5′-AGCCCCTTCTTCTCGAACTC-3′; *mMdh2* forward—5′-GAAGGCAAGCATTGAGAAGG-3′, reverse—5′-GCCCCCATGTAGCAATTAG-3′.

Polymerase chain reaction was performed on the Tercik amplifier (DNA Technology, Moscow, Russia). Real-time polymerase chain reaction (RT-PCR) was performed on the LightCycler 96 (Roche, Basel, Switzerland) using SYBR Green I dye. The parameters of amplification included an initial denaturation at 95 °C for 5 min followed by 40 cycles: 20 s at 95 °C, 30 s at 58 °C, 40 s at 72 °C, and, finally, 4 min at 72 °C. The matrix quantity was normalized relative to that of the elongation factor Ef-1ά gene [66]. Determination of relative expression of the studied genes was performed using the 2^−ΔΔC^_T_ method [67].

### 4.4. Promoter Methylation

To analyze promoters of the SDH genes *Sdh1-2*, *Sdh2-3*, *Sdh3-1*, and *Sdh4*, fumarase gene *Fum1*, and MDH genes *mMdh1* and *mMdh2* for the presence of CpG islands and selection of primers for methylation-specific PCR (MS-PCR), the UCSF program in MethPrimer-Li Lab was used (http://www.urogene.org/methprimer/index1.html, accessed on 15 June 2023) (Supplementary Tables S1–S3). Whenever possible, the primers were selected so that the analyzed CG dinucleotides were located in the area of the CpG islands. This arrangement of the analyzed cytosines made it possible to determine the dependence of the methylation status of the CpG island and its role in the regulation of expression of the analyzed genes. Determination of the methylation status of promoters of the studied genes was carried out on the basis of the results of electrophoresis of amplicons obtained using methylation-specific primers. For analysis of the nucleotide composition of promoters of the genes encoding subunits A, B, C, and D, the known sequences annotated in the GenBank were used. To determine the state of concrete CG sites in methylation-specific PCR, two versions of the forward primer differing only in the CG site were designed. In the non-methylated (U) version, cytosine was replaced by thymine, while in the methylated (M) version, cytosine was preserved. In the methylation-specific PCR, it was determined which primer was effective in each sample. Ideally, in a uniform matrix, the non-methylated and methylated primers for the same CG site should operate in the counter phase, with one operative and the other silent, and vice versa (see Supplementary Tables S1–S3 for the full set of primers).

Subsequent PCR with methyl-specific primers was performed using AmpliSence reagent (Helicon, Russia). PCR was performed using the Tercik amplificatory system (DNA Technology, Moscow, Russia). The parameters of amplification were as follows: preliminary denaturing at 95 °C for 5 min and then 35 cycles: 95 °C for 20 s, 55 °C for 20 s, 72 °C for 30 s, and finally 72 °C for 4 min. Quantification of MS-PCR was performed on the basis of electrophoretic separation of the PCR products. The extent of promoter methylation was an integral index obtained on the basis of the PCR analysis of the tested CG dinucleotides in the promoter of a particular gene. Three types of results could be expected: absence of methylation, partial methylation, or complete methylation. Therefore, we introduced the following definitions: 0% methylation—all three tested dinucleotides were not methylated; 25% methylation—1 or 2 CG dinucleotides were partially methylated; 50% methylation—1 or 2 CG dinucleotides were completely methylated; 75% methylation—1 or 2 CG dinucleotides were partially methylated, but other dinucleotides were completely methylated; 100% methylation—all three dinucleotides were methylated (for more details see [24]).

### 4.5. Statistical Analysis

The experiments were performed with three biological and four analytical repeats and the data were subjected to two-way analysis of variance (ANOVA), employing a general linear model for main effect using STATISTICA version 9 data analysis software (Statsoft Wipro, East Brunswick, NJ, USA). The data in the figures represent the means of three biological repeats ± SD. Statistically significant differences at *p* < 0.05 are discussed.

## 5. Conclusions

The dicarboxylic branch of the tricarboxylic acid cycle is regulated by light via the phytochrome mechanism at the level of the genes encoding the catalytic subunits of SDH, the mitochondrial fumarase, and one of the isoforms of mitochondrial MDH. Regulation of expression of the flavoprotein subunit of SDH and the mitochondrial fumarase involves the mechanism of promoter methylation of their genes.

## Data Availability

The datasets generated for this study are available upon request from the corresponding author.

## Supplementary Materials

The supporting information can be downloaded at: https://www.mdpi.com/article/10.3390/ijms241210211/s1.

## Abbreviations

CaM	calmodulin
MDH	NAD-dependent malate dehydrogenase
PIF	phytochrome-interacting factor
Pr	inactive form of phytochrome
Pfr	active form of phytochrome
SDH	succinate dehydrogenase
TCA cycle	tricarboxylic acid cycle
