# Supplementary material for: Light-Dependent Expression and Promoter Methylation of the Genes Encoding Succinate Dehydrogenase, Fumarase, and NAD-Malate Dehydrogenase in Maize (Zea mays L.) Leaves
Source: Int J Mol Sci. 2023 Jun 16;24(12):10211. doi: 10.3390/ijms241210211 (PMC10299040; doi:10.3390/ijms241210211)
Supplement: Supplementary file 1 [file ijms-24-10211-s001.zip › ijms-2428425-supplementary.pdf]

Supplementary Table S1. Oligonucleotides for methyl-specific PCR for the promoters of the succinate dehydrogenase genes *Sdh1-2, Sdh2-3, Sdh3-1, Sdh4*

Gene		Distance	M or U	5′–oligonucleotide–3′
Sdh1-2	I	−57	forward M	AATATATTTTAAATTGCA
			reverse M	TAATTTAAGGTTAGAGG
			forward U	AATATATTTTAAATTACA
			reverse U	TAATTTAAGGTTAGAGG
	II	−115	forward M	TAACCCACCTTACAAGCA
			reverse M	TAATTTAAGGTTAGAGG
			forward U	TAACCCACCTTACAAACA
			reverse U	TAATTTAAGGTTAGAGG
	III	−307	forward M	CAAAATAAAACTATTGCT
			reverse M	TAATTTAAGGTTAGAGG
			forward U	CAAAATAAAACTATTACT
			reverse U	TAATTTAAGGTTAGAGG
Sdh2-3	I	−109	forward M	TTTTATACGATCGAGTTAGTACG
			reverse M	AAAATATCTTTAAATAAATCTTAAACC
			forward U	TTTTTTTATATGATTGAGTTAGTATG
			reverse U	AAAATATCTTTAAATAAATCTTAAACC
	II	−165	forward M	TTAATAATATCAACAAGCG
			reverse M	TTAGTTATAAATTTTGATTG
			forward U	TTAATAATATCAACAAACG
			reverse U	TTAGTTATAAATTTTGATTG
	III	−205	forward M	AAATCTTTCTTTCACCCGCT
			reverse M	TTAGTTATAAATTTTGATTG
			forward U	AAATCTTTCTTTCACCCACT
			reverse U	TTAGTTATAAATTTTGATTG
Sdh3-1	I	−506	forward M	TATTTTTATGTTTTTTATTTTGCGG
			reverse M	TCAAGGGAACGAGTATATCTAAAC
			forward U	TATTTTTATGTTTTTTATTTTGTGG
			reverse U	TTAAGGGAATGAGTATATTTAAAT
	II	−454	forward M	TATAATAAGGGTAAGTGAAAGTCGT
			reverse M	TCAAGGGAACGAGTATATCTAAAC
			forward U	TATAATAAGGGTAAGTGAAAGTTGT
			reverse U	TTAAGGGAATGAGTATATTTAAAT
	III	−431	forward M	TTTAGAGGGGAGGTGAATAGGCGA
			reverse M	TCAAGGGAACGAGTATATCTAAAC
			forward U	TTTAGAGGGGAGGTGAATAGGTGA

			reverse U	TTAAGGGAATGAGTATATTTAAAT
Sdh4	I	−57	forward M	GATGTTTTTCGTCGTATTATTTTC
			reverse M	GTATTAGGCGGTTTTAGAGAAGG
			forward U	TGTTTTTTTGTTGTATTATTTTTGA
			reverseU	GTATTAGGCGGTTTTAGAGAAGG
	II	−115	forward M	TTTTAAAGTTTTTATTTTTTTCGA
			reverse M	GTATTAGGCGGTTTTAGAGAAGG
			forward U	TTTTAAAGTTTTTATTTTTTTTGA
			reverse U	GTATTAGGCGGTTTTAGAGAAGG
	III	−307	forward M	AAATTAGATTTAATTAATTTCGT
			reverse M	GTATTAGGCGGTTTTAGAGAAGG
			forward U	AAATTAGATTTAATTAATTTTGT
			reverse U	GTATTAGGCGGTTTTAGAGAAGG

Supplementary Table S2. Oligonucleotides for methyl-specific PCR for the promoters of the fumarase gene *Fum1*

Gene		Distance	M or U	5′–oligonucleotide–3′
Fum1	I	−495	forward M	GGGTGGATTTATATAGAGTGGGTC
			reverse M	ACGAATACCTCATACGAAATAACG
			forward U	GGTGGATTTATATAGAGTGGGTTG
			reverse U	CAAATACCTCATACAAAATAACAAA
	II	−524	forward M	AGGGGTATTACGTGTGGGTTCG
			reverse M	ACGAATACCTCATACGAAATAACG
			forward U	AGGGGTATTACGTGTGGGTTTG
			reverse U	CAAATACCTCATACAAAATAACAAA
	III	−557	forward M	TATTTATGTATAAAATTCG
			reverse M	ACGAATACCTCATACGAAATAACG
			forward U	TATTTATGTATAAAATTTGT
			reverse U	CAAATACCTCATACAAAATAACAAA

Supplementary Table S3. Oligonucleotides for methyl-specific PCR for the promoters of the NAD-malate dehydrogenase genes *mMdh1* and *mMdh2*

Gene		Distance	M or U	5′–oligonucleotide–3′
mMdh1	I	−365	forward M	CTCCTATAACTATTATCAATATAAAGCA
			reverse M	GATTATTGGTTGAGTAGGAGG
			forward U	CTCCTATAACTATTATCAATATAAAACA
			reverse U	ATTATTGGTTGAGTAGGAGG
	II	−245	forward M	AAATCTAAATAAACAATCGCC
			reverse M	GATTATTGGTTGAGTAGGAGG
			forward U	CATAAATCTAAATAAACAACA
			reverse U	ATTATTGGTTGAGTAGGAGG
	III	−194	forward M	CCGCTAAAAAAATAATAGCA
			reverse M	GATTATTGGTTGAGTAGGAGG
			forward U	CCGCTAAAAAAATAATAACA
			reverse U	ATTATTGGTTGAGTAGGAGG
mMdh2	I	−634	forward M	GTTTGTTTGTTTGTTGGTTATTGTC
			reverse M	TTCCTAACTCCCCTAAAACTCGT
			forward U	TTGTTTGTTTGTTGGTTATTGTTGT
			reverse U	TTTTCCTAACTCCCCTAAAACTCAT
	II	−704	forward M	TGTTTTGTATTTTTTTATTATCGA
			reverse M	TTCCTAACTCCCCTAAAACTCGT
			forward U	TGTTTTGTATTTTTTTATTATTGA
			reverse U	TTTTCCTAACTCCCCTAAAACTCAT
	III	−803	forward M	TTTTTTTCGTTGTTTTTTAGGTCGT
			reverse M	TTCCTAACTCCCCTAAAACTCGT
			forward U	TTTTTTTCGTTGTTTTTTAGGTTGT
			reverse U	TTTTCCTAACTCCCCTAAAACTCAT

Note: The minus sign indicates at what distance from the beginning of the first exon of the gene the studied cytosine is located; I, II, III - different groups of primers. Cytosine in each group of primers differed by the presence of methylation (M) or its absence (U).
